# Experiments on the Nerves, Particularly on Their Reproduction; and on the Spinal Marrow of Living Animals

**Published:** 1797

**Authors:** William Cruikshank


XIV. *Experiments on the Nerves, particularly on their Reproduction; and on the spinal Marrow of living Animals. By* William Cruikshank, *Esq.* Vide *Philosophical Transactions of the Royal Society of London, for the Year* 1795. Part I. 4to. London, 1795.

THE experiments described in this paper were made on the nerves of the eighth pair, or par vagum, and the intercostal nerves of dogs.

The par vagum arise from the basis of the brain, and passing out of the cranium, along with the internal jugular veins, are distributed to the tongue, œsophagus, larynx, heart, and lungs; and, running on each side of the œsophagus, may be said to terminate in the stomach, liver, and semilunar ganglion of the intercostals, below the diaphragm; from whence they are again distributed to the viscera of the abdomen.

The intercostals also arise from the basis of the brain, and passing out of the cranium, along with the carotid arteries, at first run by the the fore part of the vertebræ of the neck, ſtill adhering to the coats of theſe arteries; but having reached the cheſt, they leave theſe arteries, and run before the heads of the ribs, where, ſending off branches which paſs between the ribs, they have thence been named intercoſtals. Several of theſe branches uniting, form a trunk on each ſide, which, running forwards towards the middle of the ſpine, perforates the diaphragm, and then terminates in the ſemilunar ganglion of the intercoſtals. Theſe trunks are diſtinguiſhed by the name of the anterior intercoſtals. The original trunks continue their courſe by the ſides of the lumbar vertebræ; after which, they run before the os ſacrum, and, approaching nearer each other as they deſcend, terminate before the os coccygis, in the ganglion coccygeum impar of Walther. Their branches all go to the heart, abdominal viſcera, teſticles in men, and ovaria and uterus in women. The trunks of theſe nerves are largeſt in the neck. In the human ſpecies the two nerves of each ſide are diſtinct; but in thoſe quadrupeds which our author has examined, they are ſo intimately connected, we are told, through the whole length of the neck, as to make apparently but one nerve.

The

The intercoſtal is the ſmalleſt nerve, and adheres ſo cloſely to the other, as to be with difficulty ſeparated from it. They likewiſe have appeared to him larger in the dog, compared with his bulk, than in the human ſubject. The neck was the place in which he choſe to divide theſe nerves; it was there they could be got at with leaſt danger, a circumſtance, he obſerves, which, by rendering an experiment more ſimple, makes it conſequently more to be relied on; and, in order to put the animal to as little pain as poſſible, and make the operations ſhort, he choſe to divide both nerves at once, rather than take up time in ſeparating them, and dividing them ſingly; ſo that, inſtead of four operations on each animal, he confined himſelf to two.

We come now to the experiments deſcribed by the author.

### Experiment I.

On the 24th of January, one nerve of the par vagum, with the intercoſtal nerve, were divided, in a dog, on the right ſide. The ſymptoms, conſequent to the operation, the author author obſerves, were heavineſs, and ſlight inflammation of the right eye; breathing with a kind of ſtruggle, as if ſomething ſtuck in his throat, which he wanted to get up; ſullenneſs, and a diſpoſition to keep quiet: the pulſe did not ſeem much affected, nor did he loſe his voice in the leaſt. The unfavourable ſymptoms, it is added, did not continue above a day or two; and on the eighth day he ſeemed perfectly to have recovered.

### Experiment II.

On the 3d of February, a portion, about an inch long, of the two nerves of the oppoſite ſide, was cut out in the ſame dog: his eyes, we are told, became inſtantly red and heavy; his breathing was more difficult than in the former experiment; he was ſick, and vomited frequently; the ſaliva was increaſed in quantity, and flowed ropy from his mouth; his pulſe in the groin was about 160 in a minute; he ate and drank, however, even voraciouſly at times, and had ſtools; he never attempted to bark or howl, probably becauſe he did not feel great pain; and yet his attention, it it is remarked, was not ſo much diſengaged from internal uneaſineſs, as to be excited with ordinary cauſes from without; in breathing, the inſpirations were ſlow and deep; the expirations were attended with repeated jerks of the abdominal muſcles, as if he wanted more effectually to expel what air was contained in the lungs. On the ſeventh day after this ſecond operation, he was found dead at a conſiderable diſtance from his bed. In the dead body every thing, the author obſerves, ſeemed in a ſound ſtate, except the lungs; theſe contained little or no air; in conſequence of which they ſunk to the bottom in water, and were of a red brown colour. The inner ſurface of the trachea was exceedingly inflamed, and covered with a white fluid, in ſome places reſembling pus, in others ropy, and more of the nature of mucus. The divided nerves of the right ſide were found united by a ſubſtance of the ſame colour as nerve, but not fibrous; and the extremities formed by the diviſion were ſtill diſtinguiſhed by ſwellings, rounded in form of ganglions. The ſame appearance, we are told, had taken place with reſpect to the nerves of the left ſide; though the divided extremities ſeemed to have been full two inches apart; the uniting uniting ſubſtance was more bloody than that of the other ſide. This experiment, Mr. Cruikſhank obſerves, was made to prove that the original power of action, in the thoracic and abdominal viſcera, is independent of the nerves. As he found the nerves regenerated, a circumſtance never before noticed, it occurred to him that it might be objected to the reaſoning, that the two firſt nerves were doing their office, before the two laſt were divided; to obviate this objection, he made the following experiment:

### Experiment III.

February 19th, he divided, at one operation, the four nerves compoſing the firſt claſs, in a dog. His eyes, it is obſerved, became inſtantly dull and heavy; he tottered as he walked; foamed at the mouth; vomited two or three times; breathed with exceſſive difficulty; his inſpirations were long and deep, his expirations ſhort and ſudden, but not attended with the repeated jerks of the abdominal muſcles as in the laſt animal; he barked loud every time he threw out the inſpired air from the lungs; the pulſe was quicker than before the operation.

operation. Next morning about half after eight, Mr. Cruikſhank found him apparently dead; but on examining more attentively, perceived that he breathed ſtill, though exceedingly ſlowly; his pulſe was gone; he felt cold; and his limbs were ſtretched out. On being placed near the fire, he began in a few minutes to breathe diſtinctly, and the heart now and then gave a pulſation; in about four hours he ſeemed to have got to the ſame ſtate the operation firſt left him in, and barked at every expiration, his pulſe beating then fifty in a minute. About four in the afternoon he died, having ſurvived the operation twenty-eight hours. The lungs in the dead body were found loaded with blood, but not ſo much, Mr. Cruikſhank obſerves, as to carry them to the bottom in water. The trachea was not inflamed. The nerves of the right ſide, from which a portion had been cut out, ſeemed to have undergone little alteration; they were only a little more vaſcular than uſual, and had the rounded ſwell where they had been divided. The nerves of the left ſide, which had retracted but little, and had been only divided, had their extremities covered with a plug of coagulable lymph.

Mr. Cruikſhank ſuſpected that the reaſon of the firſt dog's dying ſo ſoon was, that none of the nerves had yet acquired the power of performing their former offices; and that, were the operations performed at a greater diſtance of time, the animal would recover. With this idea he was led to repeat his experiments, allowing a greater interval to take place between the firſt and ſecond.

### Experiment IV.

March 6th, he repeated Experiment I. on a large dog. His right eye, we are told, ſeemed inſtantly affected, looked dull and inflamed; he coughed and breathed with ſome difficulty; the ſecretions from the ſalivary glands were much increaſed; he had tremors; theſe, however, Mr. Cruikſhank attributes partly to fear, as on careſſing him they diſappeared. He ate and drank very well, and had ſtools. Moſt of theſe ſymptoms, it ſeems, continued but a few days, the eye becoming more clear, and the difficulty of breathing hardly perceptible; he vomited, but only after eating, a circumſtance, the author obſerves, which often takes place in dogs in perfect health, from devouring their food too greedily. Thus he continued for three weeks; the external wound had healed, almost by the first intention; he ate greedily, and had perfectly recovered. Mr. Cruikshank supposed the regenerated nerves might now be performing their offices.

### Experiment V.

March 27th, our author repeated Experiment II. on the same dog, but did not remove quite so much of the nerves. The animal, we are told, was stupid for a minute or two, and gaped for breath; but in a few minutes more these symptoms went off; in a quarter of an hour after he ate some boiled meat, with his usual avidity; all the symptoms of the preceding operation again took place, and in the same order. The vomiting and difficulty of breathing were rather more considerable; he ate and drank, notwithstanding, and had stools. The convulsive jerks of the abdominal muscles, which hardly took place in the last experiment, were observed in this, during expiration, but were not constant, as in the first dog. On the

15th of April he was nearly as well, it is obſerved, as before the operations, only he was leaner, and perhaps weaker, from the confinement, as well as from the operations. Mr. Cruikſhank wiſhed to ſee the ſtate of the nerves; an artery was opened in the groin, and the animal expired in a few ſeconds.

On examining the dead body, the viſcera were all, to appearance, ſound. The nerves of the right ſide, which had been divided, were found firmly united; having their extremities covered with a kind of callous ſubſtance; and this leads the author to think the regenerating nerve, like bone in the ſame ſituation, converts the whole of the ſurrounding extravaſated blood into its own ſubſtance. The nerves of the left ſide were alſo perfectly united; but the regenerated nerves were ſmaller than the original; a circumſtance which the author aſcribes to the quantity of extravaſated blood having been leſs. He obſerved too, that they did not ſeem fibrous, like original nerves; but the recollection that the callus of bone is diſſimilar to the original bone, quieted, he tells us, whatever doubts could ariſe from this circumſtance.

### Experiment VI.

April 19th, our author divided the ſpinal marrow of a dog, between the laſt vertebra of the neck and the firſt of the back. The effects of this operation were as follows: the muſcles of the trunk of the body, but particularly thoſe of the hind legs, appeared inſtantly relaxed; the legs continued ſupple, like thoſe of an animal killed by electricity; the heart, on the operation being performed, ceaſed for a ſtroke or two, then went on ſlowly and fully, and, in about a quarter of an hour after, the pulſe was 160 in a minute: reſpiration was performed by means of the diaphragm only, which acted very ſtrongly for ſome hours. The operation was performed about a quarter before twelve at noon; about four in the afternoon the pulſe was ninety only in a minute, and the heat of the body exceedingly abated; the diaphragm acting ſtrongly, but irregularly. About ſeven in the evening, the pulſe was not above twenty in a minute, the diaphragm acting ſtrongly, but in repeated jerks. Between twelve at night and one in the morning, the dog was ſtill alive; reſpiration was very ſlow, but the diaphragm ſtill acted with conſiderable force. Early in the morning he was found dead.

This operation, Mr. Cruikſhank obſerves, he performed from the ſuggeſtion of Mr. Hunter, who had obſerved, in the human ſubject, that when the neck was broke at the lower part, (in which caſes the ſpinal marrow is torn through) the patient lived for ſome days, breathing by the diaphragm. This experiment, the author adds, ſhows, that dividing the ſpinal marrow at this place on the neck, if below the origin of the phrenic nerves, will not, for many hours after, deſtroy the animal; and was preparatory to the following experiment:

### Experiment VII.

April 26th, the author divided all the nerves of the firſt claſs, in a dog. The principal ſymptoms, he obſerves, of Experiment III. took place. Soon after, he performed, on the ſame animal, the operation of Experiment VI.

 when when the ſymptoms peculiar to that operation alſo took place, whilſt thoſe peculiar to Experiment III. diſappeared. His reſpirations, we are told, were five in a minute, and more regular than in Experiment III.; the pulſe beat 80 in a minute. Five minutes after, Mr. Cruikſhank found the pulſe 120 in a minute; the reſpiration not altered. The operation was performed about two in the afternoon, and at three quarters after five, the reſpirations were increaſed to fifteen in a minute; the pulſe beating 80 in the ſame time, and very regularly: the breathing, it is remarked, ſeemed ſo free, that he had the appearance of a dog aſleep. At a quarter before eight, the pulſe beat 80, and the reſpirations were ten in a minute. At three quarters after ten, the reſpirations were eight in a minute, and the pulſe at 60. The animal heat was now exceedingly abated; heat was applied to the cheſt, after which he breathed ſtronger, and raiſed his head a little, as if awaking from ſleep. At half after twelve the breathing was ſtrong, and twelve in a minute, the heart beating forty-eight in the ſame time, ſlowly, but not feebly. He ſhut his eyelids when they were touched; ſhut his mouth on its being opened; and raiſed his head a little, but but as he had not the uſe of the muſcles which fix the cheſt, he did it, we are told, with a jerk. Between four and five o'clock in the morning, the reſpirations were five in a minute, and the pulſations of the heart exceedingly ſlow and weak. He died about ſix in the morning, having ſurvived the operation ſixteen hours.

This experiment, Mr. Cruikſhank tells us, he made from the ſuggeſtion of Mr. Hunter, with a view to obviate an objection raiſed againſt the reaſoning drawn from the three firſt experiments. It was urged, it ſeems, that though by theſe experiments he had deprived the thoracic and abdominal viſcera of their ordinary connexion with the brain, yet, as the intercoſtals communicated with all the ſpinal nerves, ſome influence might be derived from the brain in this way. This experiment removed alſo the ſpinal nerves, and conſequently this objection, the author thinks, was obviated.

As he had found, by the two laſt experiments, that dividing the ſpinal marrow in the lower part of the neck did not immediately kill, although inſtant death was univerſally known to be the conſequence of dividing it in the upper part of the neck, he expreſſed, he tells us, his furprife to Mr. Hunter, that the fpinal marrow fhould, according to modern theory, be fo irritable in the one place, and fo much lefs fo in the other.

Mr. Hunter told him, that from the time he firft obferved that men, who had the fpinal marrow deftroyed in the lower part of the neck, lived fome days after it, he had eftablifhed an opinion, that animals, who had the fpinal marrow wounded in the upper part of the neck, did not die from the mere wound; but that in dividing it fo high, we deftroyed all the nerves of the mufcles of refpiration, and reduced the animal to the ftate of one hanged; whereas in dividing it lower, we ftill left the phrenic nerves, and allowed the animal to breathe by his diaphragm. Our author conceived, that if this opinion were well founded, though dividing the fpinal marrow in the lower part of the neck does not kill inftantly, whilft the phrenic nerves are untouched; yet if the phrenic nerves were firft divided, and then the fpinal marrow in the lower part of the neck, the confequence would be the fame as if it had been divided in the upper part. With a view to afcertain this point, he made the following experiment:

### Experiment VIII.

By detaching the ſcapulæ of a dog from the ſpine, and partly from the ribs, he got at the axillary plexus of nerves, on both ſides, from behind. He ſeparated the arteries and veins from the nerves, and paſſed a ligature under the nerves, cloſe to the ſpine. He thought he could diſcern the phrenic nerves, and inſtantly divided two conſiderable nerves going off from each plexus. The action of the diaphragm, he obſerves, ſeemed to ceaſe, and the abdominal muſcles became fixed, as if they had been arreſted in expiration, the belly appearing contracted. The reſpirations of the animal, we are told, were now about twenty-five in a minute, the pulſe beating a hundred and twenty. As our author was not willing to truſt the experiment to the poſſibility of having divided only one of the phrenics, (which he afterwards found was really the caſe) and ſome different nerve inſtead of the other, after carefully attending to the preſent ſymptoms, he divided all the nerves of the axillary plexus, of each

 ſide.

ſide. The ribs, he obſerves, were now more elevated in reſpiration than before; the reſpirations were increaſed to forty in a minute, the pulſe ſtill beating a hundred and twenty in the ſame time. Finding that reſpiration went on very eaſily without the diaphragm, in about a quarter of an hour after dividing the axillary plexus of each ſide, he divided the ſpinal marrow, as in Experiment VI. All the flexor muſcles of the body now ſeemed to contract, and inſtantly to relax again; and this animal died as ſuddenly as if the ſpinal marrow had been divided in the upper part of the neck.

Mr. Cruikſhank opened the cheſt, and found the heart had ceaſed its motion; he immediately introduced a blowpipe into the trachea, below the cricoid cartilage, and inflating the lungs, imitated reſpiration. The heart now began to move again, and in about three minutes was beating ſeventy in a minute. He recollected that there was ſtill a communication between the brain and the thoracic and abdominal viſcera, that the par vagum and intercoſtals were entire, and turning to the carotids, divided the nerves. He then went on inflating the lungs as before, and the heart, which had ſtopped, began to move again, beat ſeventy in a minute, and continued to do ſo for near half an hour after the animal had ſeemingly expired. Theſe appearances, the author obſerves, were not confined to the neighbourhood of the heart; one of the perſons who aſſiſted him in the experiment, cried out once, that he felt the pulſe in the groin. Mr. Cruikſhank now ceaſed to inflate the lungs, and preſuming that he could eaſily reproduce the heart's action, allowed three minutes to elapſe. On returning to inflate the lungs, he found the heart had now loſt all power of moving; and that irritating the external ſurface with the point of a knife, did not produce the ſmalleſt vibration. He then irritated the phrenic nerves with the point of a knife, and found that the diaphragm contracted ſtrongly as often as the nerves were irritated. He next irritated the ſtomach and inteſtines, which alſo renewed their periſtaltic motions. He likewiſe irritated the par vagum and intercoſtals, about an inch above the lower cervical ganglion of the intercoſtal; and the œſophagus contracted ſtrongly through its whole length: but the heart continued perfectly motionleſs. On diſſection, he found a ſmall branch of a nerve, running down from the ſecond cervical to join the the phrenic of the right ſide, but he conſidered it as too inſignificant to have any effect on the experiment.

This experiment, Mr. Cruikſhank remarks, confirms thoſe made by Mr. Hunter, in which he recovered animals by inflating their lungs, and on which his method of recovering apparently-drowned people principally reſts. It ſhows alſo, in his opinion, that reſpiration is the prime mover of the machine; and it takes off, he thinks, whatever objections might have been raiſed, from the animals, upon which he made his experiments, having the connexion with the brain entire (as the par vagum and intercoſtals were not divided) ſince here the ſame thing took place in theſe experiments where nerves could have no effect.

